# Household Water Is the Main Source of Iodine Consumption among Women in Hargeisa, Somaliland: A Cross-Sectional Study

**DOI:** 10.1093/jn/nxab377

**Published:** 2021-10-29

**Authors:** Espen Heen, Maria Romøren, Amal A Yassin, Ahmed A Madar

**Affiliations:** Department of Community Medicine and Global Health, University of Oslo, Oslo, Norway; Department of Community Medicine and Global Health, University of Oslo, Oslo, Norway; Department of Women's Health, Manhal Hospital, Hargeisa, Somaliland; Department of Community Medicine and Global Health, University of Oslo, Oslo, Norway

**Keywords:** Iodine, urinary iodine concentration, 24-h urinary iodine excretion, total fluid intake, drinking water, women, breastfeeding, Somaliland

## Abstract

**Background:**

Iodine status surveys of women in Somaliland present widely conflicting results. Previous research indicates elevated concentrations of iodine (IQR 18–72 μg/L) in groundwater used for drinking and cooking, but the relation with iodine intake is not well characterized.

**Objectives:**

We aimed to investigate the contributions of household water iodine concentration (WIC), breastfeeding, total fluid intake, hydration levels, and urine volume on urinary iodine concentration (UIC) and excretion (UIE) over a 24-h period and to define iodine status from iodine intake estimates and median UIC, normalized to a mean urine volume of 1.38 L/d (hydration adjusted).

**Methods:**

The study sample comprised 118 nonpregnant, healthy women aged 15–69 y. All participants resided in Hargeisa, and 27 were breastfeeding. Data collection consisted of a 24-h urine collection, a 24-h fluid intake diary, a beverage frequency questionnaire, and a structured recall interview. We measured UIC and WIC in all urine and in 49 household water samples using the Sandell-Kolthoff reaction.

**Results:**

WIC ranged between 3 and 188 μg/L, with significant median differences across the water sources and city districts (*P* < 0.003). Nonbreastfeeding women were borderline iodine sufficient [hydration-adjusted median urinary iodine concentration (mUIC) 109 μg/L; 95% CI: 97, 121 μg/L], whereas breastfeeding women showed a mild iodine deficiency (73 μg/L; 95% CI: 54, 90 μg/L). There were strong correlations (ρ: 0.50–0.69, *P* = 0.001) between WIC and UIC, with iodine from household water contributing more than one-half of the total iodine intake. Multivariate regression showed hydration and breastfeeding status to be the main predictors of UIC.

**Conclusions:**

Iodine from household water is the main contributor to total iodine intake among women in Hargeisa, Somaliland. Variation in female hydration and spatial and temporal WIC may explain diverging mUIC between studies. Water sources at the extremes of low and high iodine concentrations increase the risk of subpopulations with insufficient or more than adequate iodine intake.

## Introduction

Iodine is an essential micronutrient necessary for syntheses of the thyroid hormones thyroxine (T4) and triiodothyronine (T3). Outside of a relatively narrow intake range, too little or too much iodine increases the risk of hypo- and hyperthyroidism, goiter, autoimmune thyroiditis, and neurodevelopmental impairment in young children (1).

In population assessments, urinary iodine concentration (UIC) from spot urine samples is the biomarker of choice, advocated by UNICEF and the WHO (2). UIC is theoretically determined by iodine intake from water, beverages, food, and supplements in the 4–12 h before urine collection, by nonkidney excretions of iodine (feces, sweat, breast milk), and by the dilution of urine (3, 4). A median UIC (mUIC) between 100 and 200 μg/L defines iodine sufficiency in a population of children (age 6–12 y) and nonpregnant adults (2). This range allows for considerable variation in mean 24-h urine volume (Uvol) and, subsequently, in urinary iodine excretion (UIE) per day (5, 6). Populations displaying tendencies of hypo- or overhydration cannot be compared directly since this will lead to artificially high or low mUIC values, respectively (7–9). Some reported reviews have suggested an expected mean urine volume of 1.38–1.5 L in an adult population, based on the iodine RDA of 150 μg/d and a urine excretion/intake ratio of 0.92 (5, 6, 10). The latter assumes almost complete bioavailability (fraction of ingested to available iodine in systemic circulation), which seems likely with intakes of elemental iodine/iodide and iodine from milk (11–13). Iodine covalently bound to proteins (like iodine in humic substances and some seaweeds) has a lower bioavailability of 50–85%. Iodine in typical diets has a bioavailability of 80–95% (14–18). According to the sparse literature available, the fraction of elemental iodine to bound iodine in humic substances varies across sites, with implications for bioavailability (19, 20). Complete iodine sufficiency demands that 97% of a population be above the estimated average requirement (EAR), based on the cut-point approximation (10, 21). The recommended EAR in adults is 95 μg/d. Breastfeeding women lose iodine through breast milk; hence, their EAR is 200 μg/d (6).

Until recently, the population of Somalia was assumed to be iodine deficient due to low intake of eggs, seafood, and iodized salt (22–24). In 2009, the first 2-stage, geographically clustered UIC survey of children and nonpregnant women aged 15–49 y in the 3 autonomous regions (Somaliland, Puntland, and South-Central) found, surprisingly, “more than adequate” and “excessive” iodine intake according to WHO criteria (**Supplemental Table 1**) (2, 25). Across districts, the mUIC ranged from 50 to 1000 μg/L. Iodized salt intake was only associated with the differences in districts close to the Kenyan border, and a food frequency assessment could not explain the district variation or the generally high UIC. The survey found a 3-fold difference in mUIC in households utilizing surface water compared with those using deep borehole water but did not measure iodine content in household water or investigate potential confounding factors (26).

Quite a few countries in East and North Africa, Asia, Europe, and the Americas have iodine-rich spring and groundwater, and the numbers are increasing (8, 27–34). Water analyses from Denmark and China have shown high concentrations of iodine bound in humic substances, typical of marine deposits from prehistoric times (17, 20). Somalia has a similarly widespread marine geology, and underground coal and oil discoveries in Somaliland may indicate the presence of comparable hydrogeology (35, 36).

In 2019, a new iodine survey of nonpregnant women aged 15–49 y reported similar spatial patterns of mUIC and table salt iodine content, except in Somaliland (37). In this region, an mUIC of 70 μg/L was found (thus indicating iodine deficiency), in contrast to the 224 μg/L from the 2009 survey (37). Household water iodine concentration (WIC) was measured simultaneously, with IQR between 18 and 72 μg/L (37). Interestingly, the dose–response between WIC quintiles and corresponding subgroup mUIC was not linear, but U shaped (37).

The conflicting findings called for further research. In 2011, we carried out a study of fluid intake and hydration among 160 women of varied breastfeeding status, including iodine assessments in urine and household water (38). A secondary data analysis was undertaken with the following objectives:

1. Calculate mUIC, UIE, and hydration-adjusted mUIC values, stratified by breastfeeding status, as a means of exploring the assumptions underpinning previous research findings.
2. Estimate total iodine intake/d and the iodine contribution from beverages made with household water (HHWB).
3. Analyze WIC from the main household water sources in and around Hargeisa, looking for systematic differences.
4. Estimate the associations between UIC and WIC and between total iodine intake and iodine intake from HHWB, addressing the U-shaped dose-response findings.
5. Model UIC and UIE by WIC, background, and hydration variables.

## Methods

### Setting, study design, and recruitment

Approximately 25% of the estimated 2 million female inhabitants of Somaliland (former North Somalia) are living in Hargeisa, the capital city. The old areas of the city are supplied with piped water from deep boreholes managed by the public “Hargeisa Water Agency.” The remaining inhabitants utilize water trucks, which capture groundwater adjacent to riverbeds in the vicinity. Geological maps of Hargeisa show a subsurface of Cretaceous Yesomma sandstones, Eocene Auradu limestones, and sand and gravel from younger epochs (39).

We recruited women aged 15–69 y through 50 groups (*n* = 2200) in NAGAAD (https://nagaad.org/), a network of women-empowering local organizations offering everything from literacy training in camps for internally displaced people to women's rights groups for university students. In addition, because breastfeeding women were underrepresented in NAGAAD we recruited them consecutively from 2 of the 14 mother–child health clinics in the city. We attempted to capture the range of age, socioeconomic status, education, and living location within the city, as described by the random female urban subsample in the Somaliland Multiple Indicator Cluster Survey (24).

### Exclusion of participants

Women not presenting with habitual fluid and food intake, normal fluid and energy metabolism/balance, urine collection ability, and record/recall competency were excluded (**Supplemental Table 2**). Radiocontrast agents containing iodine and radioisotopic iodine treatment were not available in Hargeisa at the time of the study. Use of mineral supplements containing >50 μg iodine was an exclusion criterion. Receipts, containers, or medicines confirmed use.

### Data collection

We developed a structured questionnaire, described elsewhere (38). The women were interviewed orally in Somali about health and reproductive history, socioeconomic status, household characteristics, and drinking and cooking water use. Weight and height were measured as described elsewhere (38, 40). Total fluid (beverage) intake (TFI) and Uvol over 24 h were obtained through a 24-h memo-tool (**Supplementary Figure 1**), a 24-h recall interview, a 24-h Uvol collection, and a beverage frequency questionnaire covering an entire year, as described elsewhere and detailed in the Supplementary Methods (38). The beverage frequency questionnaire provided data for the percentage of household water intake from beverages in comparison to TFI. A nonstratified sample of household water from a subgroup of 45 women, selected based on household availability and representing 3 important water sources for Hargeisa, was analyzed for WIC. Later, 4 additional samples were taken from water truckers and tanks to capture 1 other important source (Xaraf).

### Fieldwork

Nine bilingual female nurses from Hargeisa were trained in research ethics and evaluated in their use of the exclusion criteria, questionnaire, diaries, and measurement tools. Inter-assessor agreement showed fair reliability (**Supplemental Table 3**), as detailed elsewhere (41). The research assistants piloted the practical data collection.

The fieldwork was carried out from May to June 2011, in collaboration with leaders for each women's group. The superiors made the women feel secure and ensured good compliance. Debriefings and discussions among the entire research team were held regularly.

The research participants were individually trained to use the collection tools and diaries, and their competency was verified. During the 24-h collection, a nurse contacted the women once to strengthen compliance and was available for sorting out problems. We investigated missing, conflicting, and irregular data. Double-entry (Epidata Entry, version 3.1) error was 0.26% before corrections. The 24-h collection started on Sundays (44%), Mondays (8%), and Tuesdays (48%) not coinciding with Muslim festivals. Through the 13 d of urine collection, the mean (minimum–maximum) 24-h temperature was 25°C (24°C–26°C), and the relative humidity was 58% (52–65%).

### Laboratory analysis

The incoming temperature of the urine was 12°C–18°C. Two assessors were responsible for all of the handling and measurements. The urine samples were screened with a human chorionic gonadotropin (HcG) test. Uvol was measured by a dietary scale (Seca Culina) at 1-g precision. Urine color (Ucol) was assessed visually by use of a chart displaying 8 different dilutions, from light straw-yellow to dark green-brown. The color chart had been validated and found suitable as a hydration biomarker tool for women in all reproductive states (42, 43). A urinalysis strip (Insight Xpert, 10SW, Acon Laboratories) provided grading for urine specific gravity (Usg) by visual inspection and in accordance with the “directions for use” (**Supplemental Table 4**). All strips were read under the same lighting conditions.

Urine specimens were pipetted into vials and placed in a freezer (Westpoint 380L, model WBQ-408.MX) at −25°C within 90 min of handling. After local storage, samples were airlifted to the laboratories on ice in a pressurized cabin. Digital temperature monitoring during the 3 d of transport indicated an outer box temperature of 4°C to 0°C. No vials or tubes leaked.

Urine creatinine (Ucreat) analysis was performed by Pathcare Kenya Limited. The vials were stored at 4°C until the next day. Thawed and thoroughly mixed samples were run in duplicate on a Beckman Coulter Synchron CX5 machine with the Jaffe reaction (44). Calibration was done on the same day with Urichem 1 & 2 runs (high and low creatinine concentrations) within 2 SD of expected variability.

UIC and WIC analyses were performed in Cape Town, South Africa, at the Nutritional Intervention Research Unit. This laboratory is part of the International Resource Laboratory for Iodine (IRLI) network, which participates in the Ensuring the Quality of Iodine Procedures Program (EQUIP), run by the CDC in the United States. All tubes were defrosted within 24 h at room temperature and mixed by hand tilting, followed by a Heidolph vortex. Due to a parallel research project, a mean of 7.5 vials from each of the 118 women's urine samples and 3 vials from each of the 49 water samples were analyzed.

The method was a modified microplate application of the spectrophotometric detection of the Sandell–Kolthoff reaction, measured with a Biotek ELX808 microplate reader at 405 nm (45, 46). Samples with values above the highest standard control were diluted with distilled, deionized water filtered through a MilliQ water purifier system. Intra- and interassay CVs were 2.6% and 4.2%, respectively, with controls in the range of 85–400 μg/L.

Analyses of pure water in each of the 8 groups of empty equipment and containers showed a mean (maximum) iodine recovery of 3–8 (12) μg/L. The detection limit was 5 μg/L.

### Definitions of compound variables

Compound variables were defined as follows:

1. TFI (L/24 h) = Total volume (Σ fluid container volume × proportion drunk) of beverage intake, including plain water, during the 24-h collection.
2. Iodine intake from HHWB (μg/24 h) = WIC (μg/L) × water intake (L/24 h) × (own household water use/all water use).
3. Uvol (L) = Urine weight (kg)/Usg (L/kg) × [all urine voided (L/24 h)/urine collected (L/24 h)] × [1440 min/collection length (min)].
4. UIE (μg/24 h) = UIC (μg/L) × Uvol (L/24 h).
5. Total iodine intake (μg/24 h) = UIE (μg/24 h) × 0.90 (bioavailability) × 0.92 (UIE/total body excretion).

### Statistics

A sample size of 127 women implied an a priori precision in 24 h UIC estimates of ∼±10% (47). Descriptive statistics of the water samples indicated that 50 samples would lead to large CIs but still provide valuable and significant findings at clinically relevant WIC differences.

Our data analyses were performed with Epidata Analysis v2.2 and IBM SPSS v26. Mean individual UIC and WIC values were used in the analyses, increasing analytical precision. Data from the beverage frequency questionnaire were all recoded into frequencies/d, and individual mean daily volumes of each beverage type were calculated. Distributions were inspected visually, and skewness, kurtosis, tests of normality, and outliers were assessed. Clustering effects for key outcome variables were generally small (intracluster correlation coefficient <10%) and nonsignificant when tested with linear mixed 0-models (intercept with the clusters as a random variable). Further analyzes were performed nonclustered.

For group sample sizes >30, means were compared with an independent samples t-test, adjusted for unequal variances if necessary. For >2 comparisons, 1-way ANOVA was used. With nonnormal distributions, we used a nonparametric independent samples Mann–Whitney U test, or, for >2 samples, an independent samples difference of median test or Kruskal–Wallis test for distributional differences. Correlations were assessed with Spearman's rank order (*ρ*) to avoid inflating the values in slightly skewed outcome variables. Univariate and multivariate regression honored the assumptions of linearity, no collinearity, and residual independence, normality, and homoscedasticity. In ANCOVA, equality of regression slopes were also tested. Model building was used to assess potential predictors in univariate regression, and significant or theoretically important predictors were used in hierarchical multivariate regression, removing nonsignificant contributions stepwise. The Mahalanobis distance guided the exclusion of 3 influential points/outliers (48). CIs for medians and means of skewed distributions were estimated by bias-corrected and accelerated 1000-bootstrap samples in SPSS. The significance level (α) was 0.05.

### Ethics

Research permission from the Ministry of Health in Somaliland (MOH/3c11.00/2011) was granted on 11 Jananuary, 2011. Ethical clearance was given by the Norwegian regional ethical committee (2010/1905 REK Sør–Øst) on 15 September, 2010, and extended until 2022.

Study participants were provided with information both orally and in writing, with emphasis on the voluntary character of participation and the possibility of withdrawal at any time and without any consequences. Oral consent was observed by a third party and documented with their signature. Discretion and confidentiality were prioritized. The participants received counseling on medical issues. When untreated diseases were discovered, further investigations and 3 mo of free treatment were offered. As a gesture of appreciation, study participants were gifted $10 USD.

The ethical aspects of the study were guided by the Declaration of Helsinki (49); UNESCO, IBC on Consent (50); Nuffield Council on Bioethics research in developing countries (51); and the CIOM International Guidelines for Biomedical Research and Epidemiological Studies (52, 53).

## Results

**FIGURE 1:**
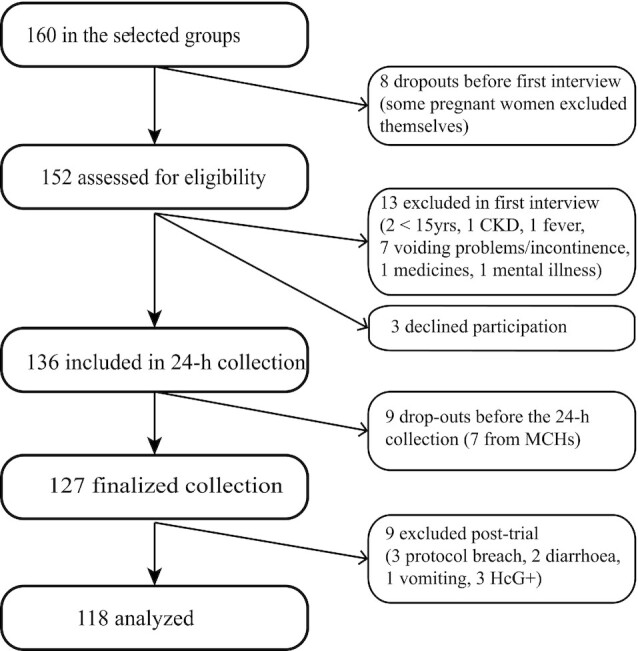
Recruitment outcome in women aged 15–69 y, with participants who were dropouts, excluded, and lost to follow-up. CKD, chronic kidney disease; MCH, mother-child health clinic.

The preselected groups comprised 160 women. After exclusion, 127 women completed the study, resulting in a participation ratio of 79% (**Figure 1**).

### Background variables

Characteristics of the women are presented in **Table 1**. The women who dropped out and who were excluded were not significantly different from those who were included in the outcome analyses (**Supplemental Table 5**). Comparison with the relevant, random MICS-4 subsample (**Supplemental Tables 6–8**) shows strong similarities.

**TABLE 1:** Background characteristics of 118 women in Hargeisa, May–June 2011^1^

	Values
Age, y	30.2 ± 12.6
Weight, kg	60.4 ± 13.6
Height, cm	161.1 ± 5.8
BMI	23.2 ± 4.8
Chronic health problem^2^	54 (45.8)
Childbirths	2.5 (0–8)
Breastfeeding	27 (22.9)
Age of breastfeeding child, mo	5 (2–12)
Fed 4–8 times	6 (22.2)
Fed >8 times	19 (70.4)
Never attended school^3^	57 (48.3)
Marital status	
Divorced/widowed	10 (8.5)
Single	48 (40.7)
Married	60 (50.8)
Male head of household	82 (69.5)
Household size	8.6 ± 3.1
Children 0–14 y in household	3.3 ± 2.3
24-h volume collection	
1 or 2 meals	15 (12.7)
≥3 meals	103 (87.3)
Diaries used correctly	114 (96.6)
Change from daily routines	13 (11.0)
Use of vitamin/mineral supplements^4^	2 (1.7)
Household salt bought at local markets	118 (100.0)
City district in Hargeisa	
Ahmed Dhagax	24 (20.3)
Mohamoud Haybe/Mohamed Mooge	35 (29.7)
Gacan Libaax	0 (0.0)
26 June	16 (13.6)
Ibrahim Koodbuur	43 (36.4)
Type of house	
Hut	32 (27.1)
Iron sheet house	37 (31.4)
Cement/brick/stone-house	49 (41.5)
Electricity in house	56 (47.5)

1 Values are means ± SDs, medians (IQRs), or *n* (%).

2 Chronic health problems were self-reported and included minor and intermittent problems.

3 Minimum schooling was defined as a 6-mo full-time course or a successful literacy course.

4 Both were using Hemotone syrup with no labeled iodine content. Their urinary iodine concentration (UIC) was close to the median value.

### Household water

Local drinking water made up 90% of TFI, with a mean consumption of 1.83 L/d (38). Water from tanker trucks, which provide deep and shallow groundwater, and water from public taps were used by 95% of the women, whereas rainwater was the primary source for 2.5%. Practically all participants (99%) used the same water source for drinking and cooking, which came entirely from their own household water supply in 96% and 98% of cases, respectively. The water source supply was described as “reliable” or “mostly reliable” in 96% of the households.

The mean and median WICs in 45 household water samples were 51 and 54 μg/L, respectively. Apart from a maximum value of 188 μg/L, the range was between 3 and 96 μg/L. There were significant (*P* < 0.0005) differences between the median WICs of the 4 water sources. The subdistributions indicated that Geed Deebleh (a borehole) displayed concentrations toward the higher values, Aw Barkhadle (a shallow well) concentrations toward the median values, Xaraf (a shallow well) concentrations toward the lower values, and Darar Weyne (a shallow well) having values spanning the entire range (**Table 2**). There were also significant differences in median WIC between the 4 city districts ( *P* = 0.003). Geed Deebleh mainly supplied women living in the Ibrahim Koodbuur and 26 June districts, whereas Darar Weyne and Aw Barkhadle supplied the Mohamoud Haybe/Mohamed Mooge district entirely. Geed Deebleh and Darar Weyne also entirely supplied Ahmed Dhagax district, where 89% of the breastfeeding women were living.

**TABLE 2:** Concentration of iodine (μg/L) in household water by 4 water sources and 4 out of 5 districts in Hargeisa, May–June 2011

Location	Mean (95% CI)^1^	Median	Minimum	Maximum	Samples, *n* (%)
Water source^2^					
Xaraf^3^	20.2 (15, 25)	20.8	13.3	26.0	4 (8)
Darar Weyne^3^	43.4 (23, 77)	28.7	3.0	187.7	11 (22)
Aw Barkhadle^3^	45.7 (29, 70)	35.5	25.5	91.7	5 (10)
Geed Deebleh^4^	65.2 (61, 70)	63.6	48.1	95.8	24 (49)
Unknown sources	33.2 (16, 57)	22.4	14.4	76.6	5 (10)
Living district^5^					
Ahmed Dhagax	40.3 (28, 51)	42.8	3.0	76.6	14 (24)
Mohamoud Haybe/Mohamed Mooge	49.8 (27, 83)	32.5	15.0	187.7	11 (28)
Gacan Libaax					0 (0)
26 June	62.7 (55, 71)	63.8	48.1	78.3	7 (14)
Ibrahim Koodbuur	68.0 (62, 75)	64.0	52.8	95.8	13 (34)

1 CI bootstrapped.

2 *n* = 49.

3 Shallow wells, often close to or in (dry) riverbeds.

4 Borehole water piped to parts of the city.

5 *n* = 45.

### Iodine intake and excretion

mUIC was 125 μg/L, and median UIE was 140 μg/d (**Table 3**). Both outcomes were significantly different between breastfeeding and nonbreastfeeding women.

**TABLE 3:** Outcome variables per 24 h, stratified by breastfeeding status, in 118 women in Hargeisa, May–June 2011^1^

Outcome	All women	Nonbreastfeeding	Breastfeeding	*P* value^2^
Participants, *n*	118	91	27	
Total fluid intake, L				0.189
Mean	2.04 (1.88, 2.20)	1.96 (1.79, 2.16)	2.29 (1.88, 2.63)	
Median	1.87 (1.64, 2.16)	1.76 (1.55, 2.07)	2.36 (1.71, 2.98)	
Min-max	0.64–4.76	0.64–4.76	0.74–4.25	
Urine volume, L				0.381
Mean	1.28 (1.17, 1.40)	1.23 (1.11, 1.34)	1.50 (1.19, 1.73)	
Median	1.15 (1.02, 1.28)	1.08 (0.99, 1.28)	1.43 (1.08, 1.81)	
Min-max	0.20–3.36	0.20–3.36	0.29–2.67	
Urine creatinine, mmol/L^3^				0.266
Mean	6.62 (5.96, 7.44)	6.44 (5.84, 7.12)	7.27 (5.33, 9.60)	
Median	5.68 (5.07, 6.36)	5.93 (5.28, 6.55)	4.76 (3.62, 7.73)	
Min-max	2.04–30.0	2.04–23.3	2.18–30.0	
UIC, μg/L				0.0005
Mean	144 (127, 164)	163 (144, 183)	81 (68, 94)	
Median	125 (113, 136)	136 (128, 144)	73 (59, 89)	
Min-max	22–781	38–781	22–147	
UIE, μg/d				0.002
Mean	162 (144, 184)	179 (156, 208)	104 (85, 123)	
Median	140 (122, 157)	151 (135, 168)	101 (78, 112)	
Min-max	31–1013	43–1013	31–222	
Total iodine intake, μg/d				
Mean	Estimates unreliable^4^	217 (193, 245)	Estimates unreliable^4^	
Median		179 (159, 203)		
Min-max		52–1223		
Total iodine intake in subsample, μg/d^5^				
Mean	Estimates unreliable^4^	193 (164, 227)	Estimates unreliable^4^	
Median		179 (159, 202)		
Min-max		73–476		
Iodine intake from HHWB in subsample,^5^ μg/d				1.00
Mean	90 (75, 107)	94 (75, 118)	82 (53, 109)	
Median	86 (64, 97)	70 (58, 96)	96 (66, 110)	
Min-max	5–317	32–317	5–141	

1 Values are presented as means or medians (95% CIs), calculated from bias-corrected and accelerated 1000 bootstrap samples. HHWB, household water beverages (beverages made with household water); Min-max, minimum—maximum; UIC, urinary iodine concentration; UIE, urinary iodine excretion.

2 *P* values describe the significance level between nonbreastfeeding and breastfeeding women and are calculated with nonparametric independent-samples differences of median test with Yates's continuity correction.

3 *n* = 116/90/26 in the 3 columns from left to right.

4 Without knowledge of iodine loss in breast milk, total iodine intake could not be estimated from UIE in breastfeeding women.

5 Subsample collected household drinking and cooking water: 40 women, 27 of them nonbreastfeeding.

**FIGURE 2:**
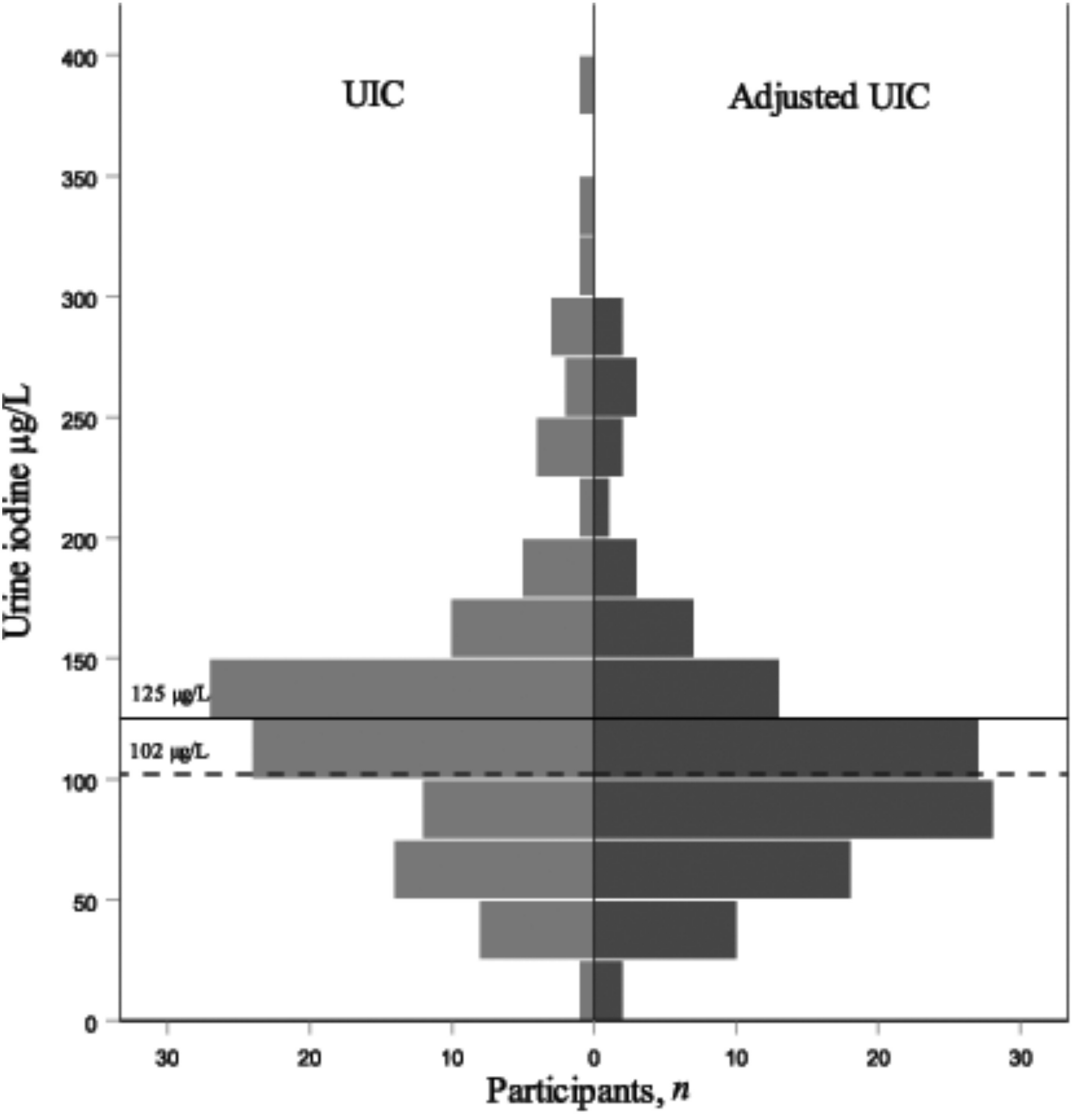
Comparison of distributions of UIC and adjusted UIC in 118 women in Hargeisa. The solid line shows median UIC of 125 μg/L, and the dashed line indicates median adjusted UIC (UIE/1.38 L) of 102 μg/L, independent-samples Mann-Whitney U test, *n* = 236, *P* = 0.002. The calculations assume that 150 μg/d of iodine intake equals a UIC of 100 μg/L with 92% of iodine ingestion excreted in urine. UIC, urinary iodine concentration; UIE, urinary iodine excretion.

**Figure 2** shows a shift in distribution from UIC to UIE due to a negative correlation between UIC and Uvol of −0.37 (*P* < 0.0005). Median UIE/1.38 L (adjusted mUIC) provides an assessment of iodine status that is comparable to mUIC in μg/L. When normalizing hydration conditions like this, mUIC decreased significantly from 125 to 102 μg/L. Median (95% CI) UIC compared with UIE/1.38 for nonbreastfeeding and breastfeeding women was 136 (126, 146) compared with 109 (97, 121) (*P* = 0.001), and 73 (54, 90) compared with 73 (49, 83) μg/L, respectively.

A 1-way between-groups (breastfeeding status) ANCOVA, adjusted for WIC in the subgroup (degrees of freedom = 40, *P* = 0.59, partial η^2^ = 0.008), retained a significant difference in estimated marginal means (95% CI) of UIC of 146 (120, 172) compared with 80 (41, 118) μg/L for nonbreastfeeding and breastfeeding women, respectively.

Nonbreastfeeding women (*n* = 90) had a median total iodine intake of 179 μg/d, with 5.5% of values below the EAR of 95 μg/d (6). Median iodine intake from HHWB was estimated to be 70 μg/d for this WIC subgroup (*n* = 27), and 38% consumed more than the EAR solely from this source. The mean percentage of iodine intake from HHWB to total iodine intake was 52% (95% CI: 43, 63). Recent studies have shown that rice, pasta, and potatoes—common Somali staple foods—can absorb considerable amounts of iodide when boiled in iodine-rich water (22, 54, 55). Adding 0.3 L household water to the estimate and assuming that ≥60% of the iodine in this water remained in the final dish regardless of preparation, the percentage of women receiving more iodine than the EAR from their household water alone increases from 38 to >50%. The largest difference in mean WIC between the districts corresponded to an iodine intake difference of 50 (95% CI: 26, 75) μg/d, estimated with the median volume of household water used for beverages. In the WIC range, household water provided a mean iodine intake of between 6 and 204 μg/d.

### Modeling of UIC and UIE

There was a strong positive correlation between WIC and UIC in the subgroup, as well as between iodine intake from HHWB and estimated total iodine intake (**Table 4**).

**TABLE 4:** Correlation analyses between iodine concentration in 40 household waters (WIC) and UIC/total iodine intake, with and without outliers, May–June 2011^1^

	Urinary iodine concentration				Total iodine intake			
	All in subgroup		Nonbreastfeeding		All in subgroups		Nonbreastfeeding	
	ρ (95% CI)	*P* value	ρ (95% CI)	*P* value	ρ (95% CI)	*P* value	ρ (95% CI)	*P* value
WIC^2^	0.50 (0.23, 0.72)	0.001	0.36	0.07	NA	NA	NA	NA
WIC^3^	0.66 (0.44, 0.83)	<0.0005	0.69 (0.34, 0.91)	<0.0005	NA	NA	NA	NA
Iodine intake from HHWB^2^	0.050	0.76	0.13	0.95	0.40 (0.12, 0.65)	0.01	0.42 (0.03, 0.70)	0.31
Iodine intake from HHWB^3^	0.20	0.24	0.27	0.21	0.47 (0.21, 0.68)	0.003	0.52 (0.22, 0.72)	0.009

1 Correlation analysis with Spearman's ρ. HHWB, household water beverages (beverages made with household water); NA, not applicable; UIC, urinary iodine concentration; UIE, urinary iodine excretion.

2 *n* = 40 in subgroup, 27 nonbreastfeeding

3 *n* = 37, 24 nonbreastfeeding. Three outliers with high UIC despite low WIC were removed from analyses.

We modeled UIC (*n* = 114) with the following independent variables: breastfeeding (yes/no), age, weight, height, BMI, TFI, WIC, and the 3 hydration measures Uvol, Ucreat, and Ucol (modeling details in Supplementary Results). The final model 1 (**Table 5**) had an adjusted R^2^ explaining 34.7% of the variability in UIC (ANOVA F = 18.46, *P* < 0.0005), with the intercept not significantly different from zero. Breastfeeding and Ucol had significant unique contributions and, when combined in a separate regression, explained 33.1% of the variability (ANOVA F = 28.73, *P* < 0.0005). Changing the model predictors to TFI, Ucreat, and breastfeeding provided an adjusted R^2^ of 0.18 (ANOVA F = 9.35, *P* < 0.0005), with all explanatory variables having unique significant contributions.

**TABLE 5:** Multivariate regression models with UIC and UIE as dependent variables in 114 women in Hargeisa^1^

Explanatory variable	Unstandardized coefficient, B (95% CI) SE	Standardized coefficient, β	t value	*P* value
Final model 1, Dv UIC				
(Constant)	−18.5 (−96.2, 59.1) 39.2		−0.47	0.637
Not breastfeeding	72.5 (41.4, 104) 15.7	0.359	4.62	0.000
Urine color, 1–8	22.6 (12.9, 32.2) 4.86	0.386	4.64	0.000
Total fluid intake, L	−16.1 (−33.0, 0.80) 8.52	−0.171	−1.89	0.062
Weight, kg	0.87 (−0.15, 1.88) 0.51	0.141	1.70	0.093
Final model 2, Dv UIE				
(Constant)	−8.72 (−64.7, 47.2) 28.2		−0.31	0.758
Weight, kg	1.38 (0.58, 2.18) 0.41	0.296	3.41	0.001
Not breastfeeding	50.6 (24.1, 77.1) 13.4	0.328	3.78	0.000

1 Dv, dependent variable; UIC, urinary iodine concentration; UIE, urinary iodine excretion.

A similar regression model using UIE as the dependent variable was explored with breastfeeding (yes/no), age, weight, height, BMI, TFI, household water intake from beverages, WIC, and Ucol as initial explanatory variables. Using WIC (*n* = 38), no model variant achieved overall ANOVA significance (0.20 < *P <* 0.42). With the full sample (*n* = 114), only weight and breastfeeding remained significant explanatory variables in the final model 2, with an adjusted R^2^ of 0.16 (ANOVA F = 11.64, *P* < 0.0005) (Table 5).

## Discussion

### Urinary iodine concentration and hydration adjustment

An mUIC of 125 μg/L indicates iodine sufficiency in women from Hargeisa, in contrast to the “more than adequate” and “iodine deficient” findings of earlier surveys (25, 37). When adjusting the mUIC value to the Uvol assumption, there is a significant reduction of 18–20%, which classifies the nonbreastfeeding women as borderline iodine sufficient. In breastfeeding women, an mUIC of 73 μg/L with the CI entirely below 100 μg/L—indicating mild iodine deficiency—does not change since the Uvol assumption is met. If ±20% represents a typical spread in median Uvol across sample populations, a hydration-adjusted mUIC in the 2009 survey could easily fall within “iodine sufficiency,” whereas the 2019 survey results would still indicate iodine deficiency. The minimum theoretical difference between the 2 surveys, based on adjusted mUIC, would be ∼100 μg/L (25, 37).

The mUIC difference related to breastfeeding status could not be explained by the covariates TFI, Uvol, Ucreat, WIC, or iodine intake from HHWB. In the regression model, breastfeeding women had 73 μg lower UIC than their nonbreastfeeding counterparts, with all other predictors equal. The obvious explanation is the extra loss of iodine through breast milk. Neither breast milk volume nor breast milk iodine concentration (BMIC) was measured in our study, and BMIC is unreliably predicted from mUIC (56, 57). An attempt to quantify breast milk volume indirectly did not generate any reliable estimates. Building on the adjusted mUIC findings, pregnant women, yet to be iodine assessed in Somalia, seem to be at high risk of iodine deficiency since they also need iodine for the growing fetus (58).

For each of the 8 steps in the Ucol scale, the model showed a UIC increase of 23 μg/L. This finding highlights how hydration is related to individual UIC concentrations and the possibility of bias when comparing mUIC values in populations with differing hydration. The lower the WIC, the more strongly this association should manifest. The preferred model was not able to explain more than 35% of the variability in UIC.

### Iodine in household water

Providing >90% of total ingested beverage and cooking water, household water was used by almost all women, thus supporting the expectation that household WIC also represents the iodine content in most fluids that the women drink (26, 37). The median WIC was in line with the 43 μg/L found in all of Somaliland in 2019 (37). The various districts of Hargeisa showed a clear preference for specific water sources, probably related to municipal water pipe distribution and the distance to the water sources utilized by tanker trucks. The maximum mean iodine intake difference of 50 μg/d, depending on the living district, is large enough to theoretically affect iodine status in subpopulations within the city and can also provide an additional explanation for the mUIC differences in the previous 2 national surveys: With large differences in WIC between adjacent districts, a limited number of women (*n* = 200), and a few villages or neighborhoods (*n* = 15–30), a lack of WIC stratification may increase the uncertainty in the point estimate (37, 59). Knowledge of fluctuating mineral concentration in underground water from the same Afro-Arabian Rift Valley complex in dry compared with wet and cold compared with hot seasons may indicate a possibility of variation in mUIC obtained during different seasons of the year (34, 60–62). These hypotheses call into question the validity of using a single mUIC value to characterize the population iodine status in all of Somaliland.

There was a strong linear correlation between WIC and UIC, contrary to the U-shaped association found in the 2019 survey (37).

### Iodine intake

In nonbreastfeeding women, the estimated median total iodine intake was 179 μg, and only 5.5% of these women were below the EAR. Habitual iodine intake distributions are usually narrower than 24-h distributions, and we may expect that virtually all nonbreastfeeding women in the study received the recommended iodine intake, of which >50% originated from household water (6). We found a strong linear correlation between iodine intake from household water and estimated total iodine intake. Breastfeeding and weight were the main predictors but did only explain 16% of the variability. We postulate that, with mean WIC between 20 and 70 μg/L, iodine intake from food and nonwater beverages constitutes the major explanatory factor in Hargeisa (38).

Iodized salt and iodine supplements seem to contribute negligibly to total iodine intake in these women. In our study, all women stated that they were buying local, non-iodized salt. The mineral supplements used by 2 women in the days before the urine collection did not contain iodine, and no woman was excluded on the basis of using iodine supplements. The results of surveys in 2010 and 2019 agreed that only 1% of household salt samples contained >15 μg iodine/g, and the mean iodine concentration was 3 μg/g (25, 37).

### Strengths and limitations

To our knowledge, this is the first study from Africa clearly linking iodine concentration in ingested household water with UIC and estimated iodine intake, using the gold standard of 24-h volume collection (63). The study design also enabled us to measure the fraction of iodine intake originating from drinking water in a free-living population. Strong linear correlations in triangulated volume data, shown elsewhere, support the integrity and accuracy of drinking and urine volumes (38). Bias in data collection was monitored extensively (38).

There are some limitations to this study. All outcome data were collected simultaneously, implying that a fraction of the UIE represented iodine ingested ahead of the 24-h iodine intake estimate. A household water batch is often stored and used for days, and with 90% of TFI originating from this source, we do not expect bias to be large. Still, these conditions affect the accuracy when comparing iodine intake from water with total iodine intake calculated from UIE and might bias effect sizes toward lower values. Iodine adsorption to and leaching from household water storage containers were not assessed. We, therefore, cannot equate the iodine concentrations in household waters with those in the source waters.

Scientific understanding of iodine absorption and regulation is still limited. In estimates of total iodine intake, we have computed a best-guess ratio between iodine intake and UIE, but the true ratio could be either larger or smaller. Humic substances, if present in household water, may have implications for bioavailability and may also increase the risk of iodine deficiency disorders through inhibition of thyroidal enzymes (19, 64).

The breastfeeding and nonbreastfeeding women who participated in this study were not random samples of all women in Hargeisa; hence, we cannot say for sure whether they represent the general population or even the same subpopulation, although there are striking similarities with the comparable, random MICS-4 subpopulation (Supplemental Tables 6–8). The reader should therefore be cautious in generalizing the absolute values to all women in Hargeisa. However, the internal relation between outcome variables in this reanalysis is valid. The homogenous iodine and fluid physiology in human beings suggests that our findings illustrate general patterns wherever there is an interplay between human hydration and/or iodine in drinking water.

## Conclusions

Hydration-adjusted mUIC values and estimates of total iodine intake characterized nonbreastfeeding women in Hargeisa as borderline iodine sufficient, whereas breastfeeding women were mildly iodine deficient regardless of adjustment. The study shows clinically relevant differences between mUIC and corresponding hydration-adjusted mUIC values. Half of the total iodine intake originated from household water. Mono–water-source preferences and a 3-fold spread in mean WIC may result in diverging iodine status in subpopulations within the city. Variation in hydration and seasonal climate, as well as nonstratification of geographical clusters with widely differing WICs, may have contributed to the opposing results in the 2 national iodine surveys of 2009 and 2019 (25, 37).

In Hargeisa, there is a need to measure hydration-adjusted mUIC in a representative sample of pregnant and breastfeeding women and to investigate if there is a difference in iodine status between the city districts. Household water sources in Somaliland need to be analyzed both spatially and temporally to map WIC, humic substances, and goitrogens, as well as to predict subpopulations at risk of iodine deficiency or excess. We recommend iodine surveys in such risk areas before any decisions are made about iodine fortification targeting the general population.

## Supplementary Material

nxab377_Supplemental_File

## Data Availability

Data described in the manuscript with the codebook and SPSS syntax will be made available upon reasonable request.

## Notes

EH and MR have been supported by 2 grants from the Norwegian Agency for Development Cooperation (Norad/Digni). Norad was not involved in any other parts of the research project.

Author disclosures: The authors report no conflicts of interest.

Supplemental Tables 1–8 and Supplemental Figure 1 are available from the “Online Supporting Material” link in the online posting of the article and from the same link in the online table of contents at https://academic.oup.com/jn/.

Abbreviations used: BMIC, breast milk iodine concentration; EAR, estimated average requirement; HcG, human chorionic gonadotropin; HHWB, household water beverages (beverages made with household water); mUIC, median urinary iodine concentration; RDI, recommended dietary intake; TFI, total fluid intake; Ucol, urine color; Ucreat, urine creatinine; UIC, urinary iodine concentration; UIE, urinary iodine excretion; Usg, urine specific gravity; Uvol, urine volume; WIC, water iodine concentration.
