# Supplementary material for: Household Water Is the Main Source of Iodine Consumption among Women in Hargeisa, Somaliland: A Cross-Sectional Study
Source: J Nutr. 2021 Oct 29;152(2):587–96. doi: 10.1093/jn/nxab377 (PMC8826834; doi:10.1093/jn/nxab377)
Supplement: nxab377_Supplemental_File [file nxab377_supplemental_file.docx]

**Household Water Is the Main Source of Iodine Consumption Among Women in Hargeisa, Somaliland: A Cross-Sectional Study**

**MD Espen Heen**

**Online Supplementary Material**

**Supplemental Table 1** Indicators of iodine status in populations adapted from (2) ^1^

|  | mUIC, µg/L | | Goiter ratio  Children  6-12 years |
| --- | --- | --- | --- |
| **Iodine status in target population** | Children 6-12 years and  nonpregnant women ^2^ | Pregnant women |  |
| **Excessive** | ≥ 300 | ≥ 500 |  |
| **Above requirements** | 200–299 | 250–499 |  |
| **Adequate/normal** | 100–199 | 150–249 | 0.0–4.9% |
| **Mild ID** | 50–99 | < 150 | 5.0–19.9% |
| **Moderate ID** | 20–49 |  | 20.0–29.9% |
| **Severe ID** | < 20 |  | ≥ 30.0% |
|  |  |  |  |

^1^ “WHO, ICCIDD, and UNICEF, Assessment of iodine deficiency disorders and monitoring their elimination. A guide for program managers. 3 ed. 2007.” ID, iodine deficiency; mUIC, mean urinary iodine concentration

^2^ Including breastfeeding women

**Supplemental Table 2** Detailed exclusion criteria for eligible women

**1) Acute disease at the time of enrollment or during the data collection**

a. Increase in frequency of defecation with nonsolid stool (diarrhea)

b. Any disturbing symptom of 1) Suprapubic pain 2) Loin pain 3) Unusual pain when voiding 4) More frequent voiding than usual 5) Urge AND 1 clear biochemical sign (2 signs, if biochemical signs alone) of nitrite, leukocytes, or blood in the urine (urinary tract infection)

c. Severe menstrual bleeding

d. Fever (more than 38.0 °C), generalized body pain, weakness, nausea, and/or vomiting affecting activities of daily living.

**2) Anorexia or nausea > 1 week at the time of enrollment**

**3) Severe chronic disease**

a. Heart disease with episodes of pitting edema (heart failure)

b. Active or end-stage liver disease

c. Subacute or chronic kidney disease with one or more of the following characteristics: Hematuria, edema, frothy urine, two or more episodes of kidney stones in the last year, glomerular filtration rate (GFR) reduced more than 50%

d. Cancer with an increased need for rest during the day compared to the pre-disease state

e. Active rheumatoid/autoimmune disease with a generalized, systemic, inflammatory reaction

f. Endocrine disorders (Addison, Cushing, or Diabetes) not treated or poorly regulated

g. Infectious disease with a generalized, systemic, inflammatory reaction

h. Difficulties with swallowing

i. Voiding problems: fistula, uterine descend, incontinence, urge symptoms, voiding duration more than 3-4 minutes

**4)** **Physical, mental or sensory impairment not compatible with taking part in the study**

**5) Medicine use**

a. Diuretics: Thiazides, loop diuretics, and spironolactone

b. Systemic glucocorticoids

**6)** **Pregnancy**

**7) Iodine supplements > 50 µg/d**

**Supplemental Table 3** Description of precision in measurements of 8 objects performed by 9 research assistants, Hargeisa 2011

| **Category** | **Measurement range^1^** | **Smallest min-max**  **diff ^2^** | **Largest min-max diff ^2^** | **Mean min-max diff ^3^** | **Mean diff in % of mean absolute measurements** | **SD ^4^** | **CV ^4^** |
| --- | --- | --- | --- | --- | --- | --- | --- |
| Weight, kg | 17.0–71.0 | 0.1 | 0.85 | 0.4 | 1.13 | 0.16 | 0.45 |
| Height, cm ^5^ | 104.2–179.0 | 0.6 | 1.4 | 1.0 | 0.66 | 0.34 | 0.23 |
| MUAC, mm | 174–295 | 6 | 12 | 9.8 | 4.0 | 3.76 | 1.53 |
| Cup volume, dL | 2.2–4.7 | 0.1 | 0.6 | 0.26 | 8.0 | 0.09 | 2.82 |

CV, coefficient of variation; MUAC, mean upper arm circumference; SD standard deviation

^1^ Shows the variation in the objects used in the measurements. Usually, the smaller the objects, the larger the imprecision.

^2^ Refers to the min-max difference (Δ) across all the assessors’ measurements on one object. For example, a min-max diff of 0.1kg shows that all the measurements performed on that object were within the range of 0.1kg.

^3^ Mean min-max differences (Δ) of all the objects within a category.

^4^ SD and CV are the mean of SD and CV for all measurements of objects within one category.

^5^ Using height as an example: The table tells us that, among the 8 objects measured, the smallest min-max difference between assessors was 0.6 cm, whereas the largest range of variation was 1.4 cm. Looking at all the measurement ranges, the average difference in height measurements was 1.0 cm. This difference constituted 0.66% of the mean height of the objects. Taking all the variation into account, the mean Standard Deviation (SD) in the measurements was 0.34 cm, and the coefficient of variation (CV) was 0.23%.

**Supplemental Table 4** Measurement options, result range, and precision for “Insight Xpert” urine dipstick

| **Analysis** | **Range** | | | | | | | **Precision (P)**  **Detection limit (DL)** |
| --- | --- | --- | --- | --- | --- | --- | --- | --- |
| U-glucose ^1^ | 0 | 1+ | 2+ | 3+ | 4+ |  |  | DL: 40mg/dL |
| mg/dL (mmol/L) | < 40 (2.0) | 50 (2.8) | 100 (5.5) | 300 (17) | 1000 (55) |  |  |  |
| U-protein ^2^ | 0 | 1+ | 2+ | 3+ |  |  |  | DL: 15mg/dL |
| mg/dL | < 15 | 30 | 100 | 500 |  |  |  |  |
| Usg ^3^ | 1 | 2 | 3 | 4 | 5 | 6 | 7 | P: +/- 0.005 |
| g/mL | 1.000 | 1.005 | 1.010 | 1.015 | 1.020 | 1.025 | 1.030 |  |
| pH ^4^ | 5 | 6 | 7 | 8 | 9 | | | |

**^1^** The Glucose test is a specific glucose-oxidase/peroxidase (GOD/POD) reaction-based method. The buffer is 3,3`,5,5` tetramethylbenzidine. It is not affected by the presence of ketones or by the pH of urine.

^2^ The semi-quantification of protein concentration uses tetrabromophenol blue in a reaction based on the principles of “protein error” of pH indicators. The test is particularly sensitive to albumin.

^3^ Usg is measured based on the apparent pKa change of certain pretreated polyelectrolytes in relation to ionic concentration. In the presence of an indicator, colors vary from deep blue to yellow-green in urine with increasing ionic concentration. Bromthymol blue and buffer are used in the reaction. The values were elevated 0.005 units if the pH of urine was ≥7 per the strip guideline.

^4^ The pH test is based on a double indicator system with methyl red sodium salt and bromthymol blue that shows the full range of pH in urine.

**Supplemental Table 5** Background characteristics of included and excluded women in the study ^1^

| **Characteristic** |  | **Included** | **Excluded/dropouts** | ***P*-value** |
| --- | --- | --- | --- | --- |
| *n* |  | 118 | 33 |  |
| Age, y |  | 30.2 ± 12.6 | 26.8 ± 10.1 | 0.15 |
| Weight, kg |  | 60.4 ± 13.6 | 67.9 ± 13.8 ^2^ | 0.12 |
| Height, cm |  | 161.1 ± 5.8 | 160.3 ± 7.0 ^2^ | 0.70 |
| BMI, kg/m |  | 23.2 ± 4.8 | 26.4 ± 5.3 ^2^ | 0.06 |
| Chronic health problem |  | 54 (45.8) | 12 (41.4) |  |
| Number of childbirths |  | 2.5 [0-8] | 2.0 [1-9] | 0.50 |
| Never attended school ^3^ |  | 57 (48.3) | 16 (48.5) |  |
| Marital status |  |  |  | 0.64 |
| Divorced/widowed |  | 10 (8.5) | 2 (6.3) |  |
| Single |  | 48 (40.7) | 11 (34.4) |  |
| Married |  | 60 (50.8) | 19 (59.4) |  |
| Male head of household |  | 82 (69.5) | 29 (87.9) | 0.06 |
| Household size |  | 8.6 ± 3.1 | 8.2 ± 2.8 | 0.73 |
| Children 0-14 years in the household |  | 3.3 ± 2.3 | 3.6 ± 2.1 | 0.43 |
| City district |  |  |  | NA ^4^ |
| Ahmed Dhagax |  | 24 (20.3) | 13 (38.2) |  |
| M. Hayb/M. Mooge |  | 35 (29.7) | 7 (20.6) |  |
| Gacan Libaax |  | 0 (0.0) | 0 (0.0) |  |
| 26th June. |  | 16 (13.6) | 5 (14.7) |  |
| Ibrahim Koodbuur |  | 43 (36.4) | 9 (26.5) |  |
| Type of house |  |  |  | NA ^4^ |
| Hut |  | 32 (27.1) | 4 (44.5) |  |
| Iron sheet house |  | 37 (31.4) | 1 (11.1) |  |
| Cement-,brick-, stone-house |  | 49 (41.5) | 4 (44.5) |  |

^1^ Values are mean ± SD, median [IQR], and *n* (%) unless otherwise indicated.

BMI, body mass index; IQR, interquartile range; NA, not applicable; SD, standard deviation.

^2^ *n* = 9.

^3^ Minimum schooling is defined as a 6-month full-time course or a successful literacy course.

^4^ Assumptions for Chi-square not met.

**Supplemental Table 6** Comparison of age, number of births, schooling, marital status, and household members between women from this study (Iodine JN) and MICS-4 (24) ^1^

| **Characteristic** | | **Iodine JN** |  | | **MICS-4 ^2^** |  |  | |
| --- | --- | --- | --- | --- | --- | --- | --- | --- |
| Study size*, n* | | 118 | |  | | |  |  |
| Woman age, y | |  | |  | | |  |  |
|  | Mean | 30.2 ± 12.6 | | 30.5 ± 13.0 | | |  |  |
|  | Median | 27 | | 26 | | |  |  |
|  | 15–19 y | 29 (24.6) | | 665 (22.3) | | |  |  |
|  | 20–24 y | 22 (18.6) | | 580 (19.4) | | |  |  |
|  | 25–29 y | 14 (11.9) | | 470 (15.8) | | |  |  |
|  | 30–34 y | 9 (7.6) | | 281 (9.4) | | |  |  |
|  | 35–39 y | 15 (12.7) | | 258 (8.6) | | |  |  |
|  | 40–44 y | 11 (9.3) | | 198 (6.6) | | |  |  |
|  | 45–49 y | 7 (5.9) | | 115 (3.9) | | |  |  |
|  | 50–69 y | 11 (9.3) | | 217 (14.0) | | |  |  |
| Births ^3^ | |  | |  | | |  |  |
|  | Mean | 3.43 | | 2.35 | | |  |  |
|  | Median | 2.0 | | 0.0 | | |  |  |
|  | 0 | 48 (44.9) | | 1206 (53.8) | | |  |  |
|  | 1 | 4 (3.7) | | 127 (5.7) | | |  |  |
|  | 2–3 | 12 (11.2) | | 243 (10.8) | | |  |  |
|  | 4–5 | 10 (9.3) | | 241 (10.8) | | |  |  |
|  | 6–7 | 12 (11.2) | | 188 (8.4) | | |  |  |
|  | 8–9 | 10 (9.3) | | 136 (6.1) | | |  |  |
|  | ≥ 10 | 11 (10.3) | | 100 (4.5) | | |  |  |
| Attended formal school | |  | |  | | |  |  |
|  | No | 79 (66.9) | | 1821 (61.6) | | |  |  |
|  | Yes | 39 (33.1) | | 1133 (38.4) | | |  |  |
|  | Primary/ALP ^4^ | 37 (31.6) | | 585 (19.8) | | |  |  |
|  | Secondary | 2 (1.7) | | 400 (13.5) | | |  |  |
|  | College/University | 0 (0.0) | | 132 (4.5) | | |  |  |
| Marital status ^3^ | |  | |  | | |  |  |
|  | Divorced/widowed | 9 (8.4) | | 173 (7.7) | | |  |  |
|  | Single | 46 (43.0) | | 1095 (49.0) | | |  |  |
|  | Married | 52 (48.6) | | 967 (43.3) | | |  |  |
| Head of household | |  | |  | | |  |  |
|  | Man | 82 (69.5) | | 1933 (64.8) | | |  |  |
|  | Woman | 36 (30.5) | | 1051 (35.2) | | |  |  |
| Household size | |  | |  | | |  |  |
|  | Mean | 8.6 ± 3.05 | | 8.2 ± 3.47 | | |  |  |
|  | Median | 8.5 | | 8.0 | | |  |  |
|  | 1 | 0 (0.0) | | 11 (0.4) | | |  |  |
|  | 2–3 | 5 (4.2) | | 188 (6.3) | | |  |  |
|  | 4–5 | 12 (10.2) | | 501 (16.8) | | |  |  |
|  | 6–7 | 26 (22.0) | | 615 (20.6) | | |  |  |
|  | 8–9 | 30 (25.4) | | 637 (21.3) | | |  | |
|  | ≥ 10 | 45 (38.1) | | 1032 (34.6) | | |  | |

^1^ Values are mean ± SD, median, and distribution *n (*%) unless otherwise indicated. UNICEF Somalia (2011). Multi Indicator Cluster Survey (MICS) 4, Somaliland, UNICEF. Data from Urban clusters in Maroodi Jeex/Sahil, where Hargeisa is the main city.

^2^ Age: *n* = 2784; Births: *n* = 2241; formal school: *n* = 2954; Marital status: *n* = 2235;

Head and household size: *n* = 2984.

^3^ Calculated from women 15-49 yrs.

^4^ In the Iodine JN study, the ALP (adult literacy program) is also defined as formal schooling.

**Supplemental Table 7** Comparison of asset wealth between women from this study (Iodine JN) and MICS-4 (24) ^1^

| **Asset wealth** | **Iodine JN** |  | **MICS-4 ^2^** |  |  |  |
| --- | --- | --- | --- | --- | --- | --- |
| Study size, *n* | 118 |  |  |  |  |  |
| Type of house |  |  |  |  |  |  |
| Cement/bricks/stone | 49 (41.5) |  | 2177 (73.2) |  |  |  |
| Other | 69 (58.5) |  | 796 (26.8) |  |  |  |
| Main material of the dwelling floor | |  | |  |  |  |
| Earth/Sand | 16 (13.6) |  | 319 (10.7) |  |  |  |
| Wood planks | 0 (0.0) |  | 21 (0.7) |  |  |  |
| Palm/grass | 1 (0.8) |  | 11 (0.4) |  |  |  |
| Ceramic tiles | 16 (13.6) |  | 759 (25.5) |  |  |  |
| Cement | 38 (32.2) |  | 1746 (58.7) |  |  |  |
| Other | 47 (39.8) |  | 120 (4.0) |  |  |  |
| Type of fuel for cooking |  |  |  |  |  |  |
| Electricity | 0 (0.0) |  | 75 (2.5) |  |  |  |
| LPG | 1 (0.8) |  | 35 (1.2) |  |  |  |
| Kerosene | 0 (0.0) |  | 15 (0.5) |  |  |  |
| Charcoal | 114 (96.6) |  | 2808 (94.2) |  |  |  |
| Wood | 3 (2.5) |  | 46 (1.5) |  |  |  |
| Straw/shrubs/grass | 0 (0.0) |  | 2 (0.1) |  |  |  |
| Asset possession |  |  |  |  |  |  |
| Electricity | 56 (47.5) |  | 2367 (79.3) |  |  |  |
| Radio | 58 (49.2) |  | 1685 (56.5) |  |  |  |
| Television | 49 (41.5) |  | 1994 (66.8) |  |  |  |
| Non-mobile phone | 8 (6.8) |  | 909 (30.5) |  |  |  |
| Refrigerator | 12 (10.2) |  | 685 (23.0) |  |  |  |
| Stove | 115 (97.5) |  | 2749 (92.1) |  |  |  |
| Wheelbarrow | 40 (33.9) |  | 706 (23.7) |  |  |  |
| Mats | 111 (94.1) |  | 2672 (89.5) |  |  |  |
| Thermos | 115 (97.5) |  | 2874 (96.3) |  |  |  |
| Kerosene lamp | 61 (51.7) |  | 1455 (48.8) |  |  |  |
| Mobile phone | 89 (75.4) |  | 2699 (90.4) |  |  |  |
| Own the house | 90 (76.3) |  | 2002 (67.1) |  |  |  |

^1^ Values are *n (*%) unless otherwise indicated. UNICEF Somalia (2011). Multi Indicator Cluster Survey

(MICS) 4, Somaliland, UNICEF. Data from Urban clusters in Maroodi Jeex/Sahil, where Hargeisa is the main city.

^2^ Type of house: *n* = 2973; floor: *n* = 2976; fuel: *n* = 2981; assets: *n* = 2984

**Supplemental Table 8** Comparison of household water characteristics between women from this study (Iodine JN) and MICS-4 (24) ^1^

| **Characteristic** | **Iodine JN** | **MICS-4 ^2^** |  |
| --- | --- | --- | --- |
|  |  |  |  |
| Study size*, n* | 118 |  |  |
| Main source of drinking water |  |  |  |
| Piped into dwelling | 35 (29.7) | 1283 (43.1) |  |
| Piped into compound | 0 (0.0) | 163 (5.5) |  |
| Piped to neighbor | 15 (12.7) | 311 (10.4) |  |
| Public tap/standpipe | 20 (16.9) | 252 (8.5) |  |
| Rainwater collection | 3 (2.5) | 21 (0.7) |  |
| Tanker truck | 42 (35.6) | 695 (23.3) |  |
| Cart with small tank/drum | 3 (2.5) | 244 (8.2) |  |
| Bottled water | 0 (0.0) | 0 (0.0) |  |
| Other ^3^ | 0 (0.0) | 8 (0.3) |  |
| Drinking water purified | 12 (10.2) | 496 (16.6) |  |
| Same water source for drinking and cooking | 117 (99.2) | 2984 (100.0) |  |

^1^ Values are *n (*%) unless otherwise indicated. UNICEF Somalia (2011). Multi Indicator Cluster Survey

(MICS) 4, Somaliland, UNICEF. Data from Urban clusters in Maroodi Jeex/Sahil, where Hargeisa is the main city

^2^ Main source and drinking water: n = 2977

^3^ Well/Borehole; Protected well; Unprotected well; Protected spring; Unprotected spring; Surface water; Other.

**Supplementary Methods**

Fluid intake and voiding frequency and volumes during the 24-h collection were obtained through four interconnected methods (I-IV). A fluid-specific diary and a beverage frequency questionnaire (BFQ) were developed, with the support of students from the University of Hargeisa, College of Medicine and Health Science. These were later piloted with illiterate women.

**(I)** Illiterate women used a 24-h **“box-diary”**— a small box with 2 zip bags containing 20 standardized, elongated blue paper strips and 20 yellow paper balls. For each serving, 1 strip was put into the box. The length corresponded roughly with how much fluid had been drunk, e.g., drinking half a cup corresponded to a half-length strip. Similarly, for each void, 1, 2, or 3 balls for small (<150ml), medium (150-300ml), or large (>300ml) urine volumes, respectively, were collected as a triangulation of Uvol. The Pearson r correlation was 0.87.

The literate women used a **“symbol-diary”** with 6 time-intervals covering 24 hours. Four different “calls for prayer” and 1 cultural snack time divided the intervals. Drinking volumes were recorded by proportional coloring of empty “symbol cups.” Coloring open “drops” within markings of 1/3, 2/3, and 3/3 indicated the volume (size) of each void, similar to the levels for the “box-diary.” The women colored extra drops if voiding more than twice within a time interval.

**II)** **In a 24-h recall interview,** each paper strip, paper ball, or colored symbol (**Supplementary figure 1**) had to be accounted for within each time interval. We investigated and corrected incomplete or erroneous use of diaries. Fluid volumes were coded with the proportions 1/1, 3/4, 2/3, 1/2, 1/3, 1/4, or 1/8 of each beverage container. The volumes of the containers used during the 24-h collection were measured to the nearest 10 ml. For bottles, the labeled volume was used. The women could also identify the actual containers from pre-measured models displaying a predominant collection of cups, glasses, and bottles for water and soft drinks available in Hargeisa. If any of this failed, they were asked to give an estimate of the container’s full volume in units of ‘dare,’ a standard size cup used for measuring milk at markets, equaling approximately 220 ml. A full drinking container was defined with a fluidless rim of 0.5-0.7cm.

**III) The Beverage Frequency Questionnaire** (BFQ) investigated how often women were drinking plain water (15 types and sources), hot drinks (4 types), milk and dairy products (6 types), juices (4 types), and soft drinks. Other alternatives could be specified. Based on the last year, a semi-quantitative number of servings of each fluid type was recorded with the alternatives: Never or less than 1 per month, 1-3 times per month, 1 per week, 2-3 per week, 4-6 per week, 1 per day, 2-3 per day, 4-6 per day, ≥ 7 per day.

**IV)** **Urine collection** of 24-hour duration (Uvol) was accomplished by providing each woman with soap, tissue paper, a plastic bag, a small towel, a beaker with marks at 150 and 300 mL, a funnel, a 500 mL bottle, and a large urine container of 2.5 L (Ashut Engineers Limited, Nairobi). The latter was designed to mimic the yellow cooking-oil cans in regular sale. Every woman received a large women’s bag, in which the smaller equipment could be hidden when she collected urine outside of her dwelling. This setup enabled the women to continue with their daily activities during the urine collection. The urine container was stored in a cool, dark place between voids.

**Supplementary Figure 1**


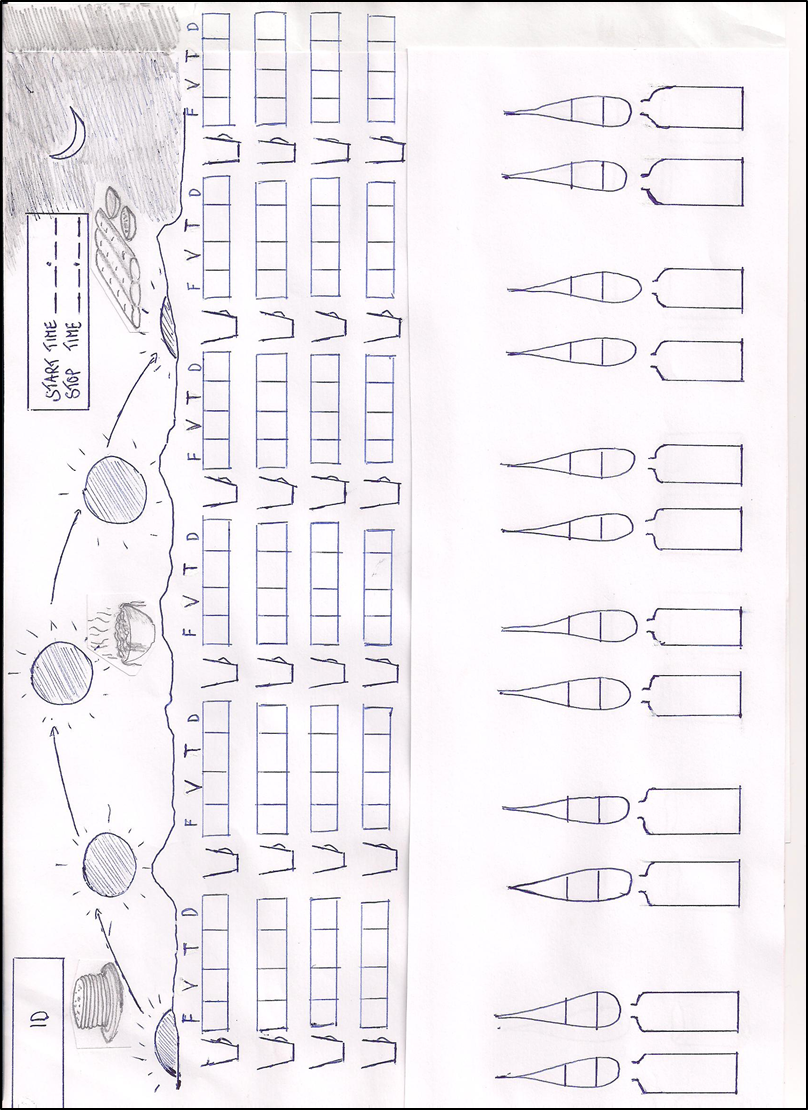


**Symbol diary in the 24-h data collection**. The women colored the open cups (beverage) and elongated drops (urine voids). The adjacent labels were used during the final interview. F = proportion that was drunken, V = direct measurement of full drinking container, T = model container volume, D = local volume unit “Dare”. The product of proportion and container volume equaled the volume that was drunken for each serving.

**Supplementary Results**

**Modeling prediction of UIC - model 1**

We modeled the prediction of UIC in µg/L (*n* = 114) with the independent variables: Breastfeeding (dummy yes/no), age, weight, height, BMI, TFI, WIC, and the three hydration measures Uvol, Ucreat, and Ucol. TFI was highly correlated with household water intake from beverages (r = 0.95) and was preferred for ease of measurement. Theoretically, TFI has a positive linear component (iodine load) and an inverse component (as fluid intake increases, urine becomes diluted and UIC values fall). In curve estimation, the inverse component was not significant; however, the slope of the linear component was not significant from zero either, possibly indicating that the hydration effect and the iodine contributing effect were canceling each other out at the WIC levels present in the sample. Weight, height, BMI, and age were not significantly different from the intercept alone in univariate regression. We retained weight as the best alternative. The other variables were reasonably linear with UIC and had a significant (Pearson) correlation between 0.3 and 0.6 and low collinearity (r < 0.75). In hierarchical regression, Ucol was the best hydration variable, with Uvol and Ucreat not adding significantly to the model. Three outliers were removed because the Mahalanobis distance was > 25. WIC in the model provided significant overall ANOVA, but not a significant individual effect size, although unstandardized B was large, indicating that, for every 1 µg/L increase, the predicted UIC would increase with 0.68 µg/L. Compared to the final model 1, the maximally adjusted R square was 0.30. We expect the main problem to be the low sample size (*n* = 38). The final model 1 is presented in table 5 in the article, with an adjusted R square explaining 34.7% of the variability in UIC (ANOVA F = 18.46, p < 0.0005) and with the intercept not significantly different from zero. Breastfeeding and Ucol had significant unique contributions and, when combined in a separate regression, explained 33.1% of the variability (ANOVA F = 28.73, p < 0.0005). Changing the model predictors to TFI, Ucreat, and breastfeeding provided an adjusted R square of 0.18 (ANOVA F = 9.35, p < 0.0005); these were all predictors with unique significant contributions. Ucreat was not associated with weight, height, or BMI in univariate regression (results not shown).
